# The Effect of Cerebrolysin on Anxiety, Depression, and Cognition in Moderate and Severe Traumatic Brain Injury Patients: A CAPTAIN II Retrospective Trial Analysis

**DOI:** 10.3390/medicina58050648

**Published:** 2022-05-09

**Authors:** Ioana Anamaria Mureșanu, Diana Alecsandra Grad, Dafin Fior Mureșanu, Elian Hapca, Irina Benedek, Nicoleta Jemna, Ștefan Strilciuc, Bogdan Ovidiu Popescu, Lăcrămioara Perju-Dumbravă, Răzvan Mircea Cherecheș

**Affiliations:** 1 RoNeuro Institute for Neurological Research and Diagnostic, No. 37 Mircea Eliade Street, 400354 Cluj-Napoca, Romania; diana.grad@ssnn.ro (D.A.G.); dafinm@ssnn.ro (D.F.M.); elianhapca@gmail.com (E.H.); dr.irinabenedek@gmail.com (I.B.); niko_j2005@yahoo.com (N.J.); stefan.strilciuc@ssnn.ro (Ș.S.); 2 Department of Neurosciences, “Iuliu Hatieganu” University of Medicine and Pharmacy, 400012 Cluj-Napoca, Romania; lperjud@gmail.com; 3 Department of Public Health, Babes-Bolyai University, No. 7 Pandurilor Street, 400376 Cluj-Napoca, Romania; razvan.m.chereches@gmail.com; 4 Department of Neuroscience, Carol Davila University of Medicine and Pharmacy, 050474 Bucharest, Romania; bogdan.popescu@umfcd.ro

**Keywords:** Cerebrolysin, traumatic brain injury, post-TBI depression, post-TBI anxiety, post-TBI cognitive impairments

## Abstract

*Background and Objectives:* Traumatic brain injuries represent an important source of disease burden requiring emergency inpatient care and continuous outpatient tailored rehabilitation. Although most TBIs are mild, patients are still developing post-TBI depression, anxiety, and cognitive impairments. Our secondary retrospective trial analysis aimed to (1) analyze correlations between HADS-Anxiety/HADS-Depression and scales that measure cognitive and motor processes in patients treated with Cerebrolysin compared to the placebo group and (2) compare anxiety and depression scores among the two treatment groups. *Materials and Methods:* Our secondary retrospective analysis focused on TBI patients with moderate and severe disability divided into two groups: Cerebrolysin (treatment) and saline solution (procedural placebo). We analyzed data from 125 patients. We computed descriptive statistics for nominal and continuous variables. We used Spearman’s correlation to find associations between HADS and other neuropsychological scales and the Mann–Whitney U test to compare HADS-Anxiety and HADS-Depression scores among the two study arms. *Results:* Our sample consisted of patients with a mean age of 45.3, primarily men, and with a 24 h GCS (Glasgow Coma Scale) mean of 12.67. We obtained statistically significant differences for HADS-Anxiety during the second and third visits for patients treated with Cerebrolysin. Our results show that Cerebrolysin has a large effect size (0.73) on anxiety levels. In addition, there are positive and negative correlations between HADS-Anxiety and Depression subscales and other neuropsychological scales. *Conclusions:* Our secondary database analysis supports the existing body of evidence on the positive effect of Cerebrolysin on post-TBI mental health status. Future confirmatory trials are necessary to clarify the link between the intervention and measured outcomes.

## 1. Introduction

Traumatic brain injuries (TBIs) are an enormous source of preventable disability and mortality, affecting young children, adolescents, adults, and the elderly alike [1,2,3]. Studies focusing on TBI epidemiology have identified people under 25 and over 75 years as the most affected [4]. The rate of TBI cases, TBI-related deaths, and admissions to the ICU (intensive care unit) were higher among men than among women [5,6]. The leading cause of TBI differs across country income classification, with falls being predominant in high-income countries (HICs) and road traffic accidents in low- and middle-income countries (LMICs) [7]. These patterns are due to an increased proportion of older adults in HICs, unsafe road infrastructure, and a lack of adherence to safety measures in LMICs [7,8]. As it is estimated that by 2050 over 80% of the older adults will reside in LMICs, a socioeconomically driven shift in TBI-related causes is likely to occur [8].

TBI, characterized as “an alteration in brain function, or other evidence of brain pathology caused by an external force”, has caused over 27 million cases and 8.1 million years lost due to disability (YLDs) globally, in 2016 [9,10]. Romania was the second most affected country in Central Europe (one of the regions with the highest numbers of TBI cases at the global level) in 2016, with 169,215 cases. In the European Union, it is estimated that out of 490,000 new cases per 100 million population, more than half required hospital admission, and approximately 11,000 resulted in death [7,10]. Post-TBI, patients are at increased risk of suffering from depression (with rates between 6 and 77%) and anxiety (rates vary across studies from 3.7% to 50%) [11,12,13].

TBI clinical severity is routinely evaluated using the Glasgow Coma Scale (GCS), an instrument widely used in research and clinical practice. Based on three domains (eye, motor, and verbal), GCS classifies TBI as mild (13–15), moderate (9–12), or severe (3–8). Nine out of ten diagnosed TBIs are classified as mild [14]. However, regardless of TBI severity, patients can still experience a wide range of short- and long-term psychological (i.e., anxiety, depression, post-traumatic stress disorder), physical, and functional (i.e., difficulties in reaching, lifting, arising from a chair, and activities related to daily living) impairments, impacting patient quality of life and hence leading to a significant burden to society [12,15,16,17].

Cerebrolysin is a biological agent used to treat several neurological diseases, including traumatic brain injury, vascular dementia, and ischemic stroke [18]. Clinical studies have shown the efficacy of Cerebrolysin as measured by improved functional recovery and decreased levels of depression in moderate and severe TBI [19,20].

This study based on secondary data analysis aimed to (1) analyze correlations between HADS (Hospital Anxiety and Depression Scale)-Anxiety and -Depression subscales and scales that measure cognitive and motor processes in TBI patients treated with Cerebrolysin compared to patients from the placebo group and (2) compare anxiety and depression scores among the two treatment groups.

## 2. Materials and Methods

We analyzed secondary, retrospective data obtained from the CAPTAIN II study. The placebo-controlled, double-blind, randomized clinical trial compared a multidimensional ensemble of clinical and neuropsychological outcomes in patients with moderate and severe TBI treated with Cerebrolysin. In total, 142 TBI patients were included between 24 April 2013 and 28 December 2017, with 90 days since the TBI for each patient. The randomization ratio was 4 (Cerebrolysin) to 3 (procedural placebo) patients based on a Research Randomizer code. The randomization procedure (defined within a randomization plan) took place in a validated space located in the Cluj-Napoca Emergency County Hospital. The process of treatment allocation was blinded, and patient block size was not available in the trial protocol, thus removing a potential source of bias. The study protocol was approved by the local Ethics Committee within the Iuliu Hatieganu University of Medicine and Pharmacy, Cluj-Napoca (No. 714/07.03.2013), and may be retrieved from the ISRCTN registry (No. 1709716) [21].

Patients with isolated TBI were eligible based on the following criteria listed in Table 1.

Patients were divided into two study groups: Cerebrolysin (intervention) and saline solution (procedural placebo). After patients were screened and, based on the checklists, included or excluded, assessments were carried out at three time points: day 10, 30, and 90. Three treatment courses were administered based on the following calendar: between days 1 and 10, 31 and 40, and 61 and 70. Patients from the intervention group received 50 mL of Cerebrolysin for the first treatment course and 10 mL of Cerebrolysin for the second and third ones, diluted in 250 mL of 0.9% NaCl before administration. The placebo group received 250 mL of 0.9% NaCl for all courses.

In addition to laboratory results, patients were also subjected to cognitive evaluations during the three study visits.

For our analysis, we selected six scales used to measure primary outcomes and one (Finger Tapping Test) for secondary outcomes. Assessments were carried out by neuropsychologists or medical doctors trained to use neuropsychological measurements (Table 2).

Additional information on other collected variables during CAPTAIN II study has been thoroughly described in past publications [20,29].

### Statistical Analyses

Descriptive statistics were compiled to characterize our study sample (i.e., mean and standard deviation for continuous variables, counts and percentages for nominal/dichotomous variables).

We used Spearman’s correlation to determine associations between the scores of HADS-Anxiety and -Depression and Color Trails Test, Stroop Color–Word Test—Victoria Version, Digit Span, and Mini-Mental State Examination at 30 and 90 days after the TBI.

A Mann–Whitney U test was used to establish group score differences for HADS-Anxiety and -Depression subscales between TBI patients treated with Cerebrolysin and TBI patients from the placebo group at the second and third study visits. Study hypotheses are formulated in Table 3.

Assuming a similar distribution of the two groups of the independent variable (treatment), the Mann–Whitney U test was used to infer a statistically significant median difference between the dependent variables (HADS-Anxiety and -Depression subscale scores). Alternatively, the Mann–Whitney U test assessed statistically significant differences in the mean ranks for anxiety and depression subscales scores among the two study groups [30]. The effect size for Mann–Whitney U was computed using an online calculator [31]. Analyses were conducted using SPSS version 26 (IBM, US) [32]. The alpha level threshold was set at 0.05.

## 3. Results

We analyzed data from 125 patients (71 from the treatment group and 54 from the control group) out of the initial 142 eligible enrolled patients. We excluded 17 patients (9 from the treatment group and 8 from the placebo group) from the analysis due to missing data. The mean age of the sample was 45.3 years (SD: 16.75, range: 18–79). The GCS score was 11.06 (SD: 1.19) before treatment administration and 12.67 (SD: 1.43) twenty-four hours after admission. Out of all patients, 88.88% were male, and 26.4% were between 31 and 40 years (Table 4). Descriptive statistics (mean and SD) for each scale we included in our analysis are presented in Table 5.

The most common causes of traumatic brain injury were falls from another level (*n* = 40) and traffic accidents (*n* = 30), while the least frequent causes were falling from a bicycle, being hit by a blunt object, and assault (*n* = 1).

**Figure 1:**
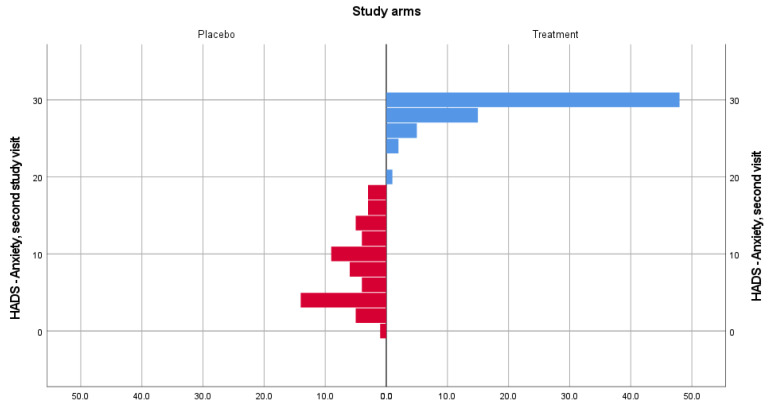
HADS-Anxiety score distribution for the treatment and placebo groups for the second visit.

**Figure 2:**
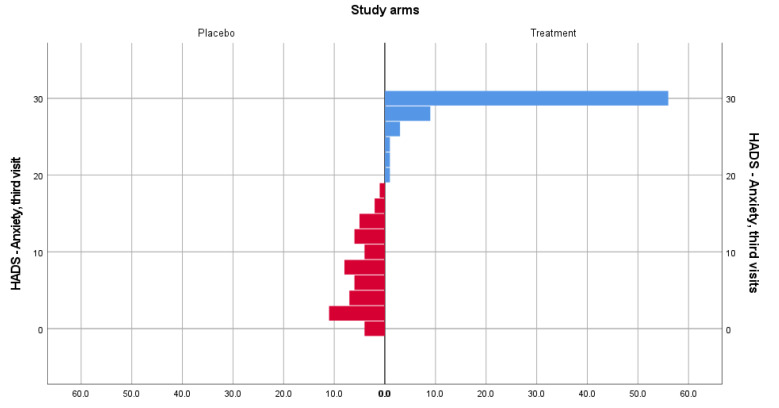
HADS-Anxiety score distribution for the treatment and placebo groups for the third visit.

In the case of the HADS-Anxiety subscale, distributions of the scores were not similar. However, there was a statistically significant difference in anxiety scores both at the second (U = 3834, z = 9.567, *p* = 0.000) and third (U = 3834, z = 9.792, *p* = 0.000) visits for patients included in the treatment group (mean rank = 90) and placebo group (mean rank = 27.50). Figure 1 and Figure 2 show data distribution among patients from both groups, for visits 2 and 3.

**Figure 3:**
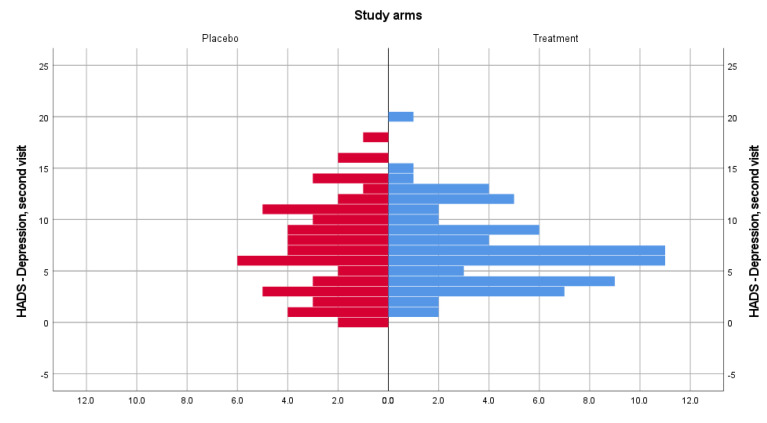
HADS-Depression score distribution for the treatment and placebo groups for the second visit.

**Figure 4:**
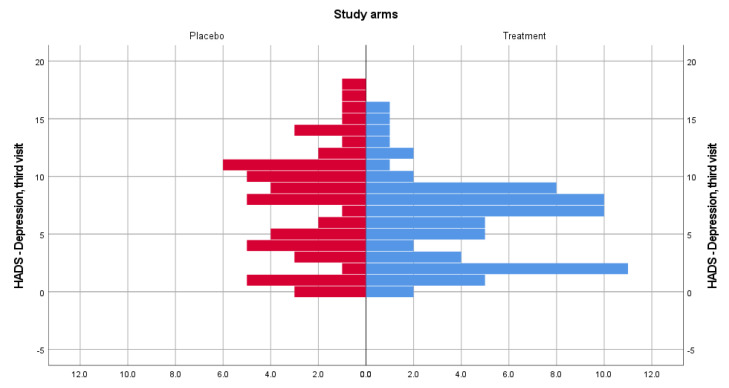
HADS-Depression score distribution for the treatment and placebo groups for the third visit.

For the HADS depression subscale, the distributions of scores were similar. Median depression scores for patients from the treatment group (median visit 2, visit 3 = 7) and placebo group (median visit 2 = 7, median visit 3 = 8) were not statistically significant different at the second (U = 1906.5, z = −0.053, *p* = 0.958) and third (U = 1547, z = −1.850, *p* = 0.065) study visits. Figure 3 and Figure 4 show data distribution among patients from both treatment groups, for visits 2 and 3. Based on computed effect size, Cerebrolysin has a large effect size on anxiety at both visits (0.73) and a small effect on depression at visit 3 (0.023).

Correlations of HADS-Anxiety and -Depression subscales with other neuropsychological scales are presented in Table 6 and Table 7. Statistically significant negative correlations at the third study visit are reported between HADS-Anxiety and FFT (Left and Right), PSI, and Digit Span Forward and a statistically significant positive correlation between HADS-Anxiety and CCT2, Stroop W-D, PSI, and MMSE.

## 4. Discussion

Our retrospective study aimed to compare the effect of Cerebrolysin on anxiety and depression among clinically classified patients with moderate or severe TBI. We report statistically significant improvements in anxiety levels measured at the second and third study visits. Our secondary analyses show that Cerebrolysin has a large effect on anxiety levels but a small one on depression levels. Our results support previously published findings, confirming that patients randomized to the Cerebrolysin group exhibit better outcomes than patients randomized to the control group [29]. Male patients predominantly form our study cohort. This result is in line with other articles that state that most TBI cases and hospitalizations are among men [6,33].

Although our results align with previously published findings, our analysis also yielded some diverging results, requiring further research. One of our main variables of interest was HADS (Hospital Anxiety and Depression Scale). The HADS score is precise in evaluating depression and anxiety symptoms, and it is a predicting factor for the clinical diagnosis of anxiety or depression at twelve months following a medium or severe TBI (considering the DSM IV diagnosis criteria) [34].

Compared to other trials studying the effect of neuroprotective agents in TBI patients, the CAPTAIN trial series employed a multidimensional approach to capture the global impact of a traumatic brain injury (on cognitive, neuropsychological, and physical functioning). In the original CAPTAIN II, authors reported statistically significant results among patients treated with Cerebrolysin (at an interval of 90 days after study inclusion) for the following neuropsychological tools: Digit Span Forward (*p* = 0.0304) and Backward (*p* = 0.0122), Stroop Word/Dots Inference (*p* = 0.0073), and HADS-Depression subscale (*p* = 0.0122) [29]. Our results are partially similar for Digit Span Forward (for both visits) and Digit Span Backward (at the second visit), while our analysis reports statistically insignificant results for HADS-Depression. The differences reported above can be explained by a variation in the sample size and treatment of missing data. The mentioned article analyzed data from 139 patients. Depending on the cause of missing data, strategies such as worst rank imputation (lack of collected data as a result of patient’s death or due to post-TBI neurological outcomes), Last Percentile Carried Forward (other reasons than TBI or additional injuries in other body regions), and Last Observation Carried Forward (for patients with an increased level of disability recorded using GOSE at the first and third visit) were integrated into the data analysis plan [29]. Our choice of sample size and treatment of the missing data of outcome scales was driven by the objective to evaluate the association between the neuropsychological tools and group differences between patients from the two study cohorts, rendering a smaller sample size.

CAPTAIN I, a two-stage parallel cohort, randomized multi-centric trial evaluated clinical efficacy and safety as an add-on therapy for moderate and severe TBI patients hospitalized in several countries in the Asia-Pacific region. This trial showed that Cerebrolysin reduced the burden of anxiety and depression (*p* = 0.0438). However, contrary to our findings, a large effect was reported on the HADS depression subscale (MW = 0.72). In addition, 61% of patients treated with Cerebrolysin (compared to 41% of patients from the placebo group) had decreased overall scores for HADS [35].

A meta-analysis using individual patient data from CAPTAIN I and II reports a medium-sized effect (MW > 0.64, *p* < 0.01) of Cerebrolysin on depression. Out of the 188 patients included in this prospective meta-analysis, a score between 0 and 7 for HADS (at day 90) was observed in 70.5% of the included patients from the treatment group compared to 39.5% of patients from the placebo group [35]. Another meta-analysis focusing on the efficacy of Cerebrolysin showed that functional recovery was registered among TBI patients with varying severity diagnoses [19].

Khalili and colleagues conducted a single-center study and included patients scoring 2 or 3 on the GOS one month after the TBI event. Most cases were related to road traffic accidents (53.8% were involved in a motor vehicle accident while 27.6% were pedestrians). The included participants (out of 129, only 65 were administered the treatment) received 10 mL of Cerebrolysin diluted in 100 mL of saline solution for 30 days. Based on study evaluations, patients with severe disability post-TBI included in the treatment group had (statistically significant) increased GOS-E scores when analyzing the reported values for baseline and at six months and three and six months compared to patients from the control group. In terms of mortality levels, 23.6% more deaths were reported among patients from the control group [36].

Improvement in functional outcomes following a TBI plays a vital role in patient recovery as patients with low scores of GOSE are diagnosed with depression and PTSD and report decreased levels of quality of life at twelve months post-TBI [37].

The mean age of TBI patients included in studies evaluating the effect of Cerebrolysin varies from 33.3 to 47.4 years, while the percentage of male participants ranges from 56% to 88.5%. Other differences were reported for treatment initiation, duration, and doses [20,29,35,36].

Cerebrolysin has also been used as a pharmacological add-on in patients diagnosed with stroke, vascular dementia, and Alzheimer’s disease [38,39,40]. In studies that included patients with vascular dementia, a beneficial effect was observed on cognitive function after treatment with Cerebrolysin [39]. Cognitive and functional improvements have been reported for patients diagnosed with Alzheimer’s disease dementia (in different stages of dementia severity) [41].

## 5. Conclusions

Cerebrolysin is currently used as an adjunctive treatment in patients diagnosed with stroke, vascular dementias, and Alzheimer’s disease [38,39,40]. While supporting the existing literature on the positive effect of Cerebrolysin on mental health after TBI, our secondary analysis indicates the need for additional confirmatory studies (e.g., a high-quality, large-sample clinical trial) to establish clear relationships between the agent and post-traumatic mental health outcomes, as well as across multiple measurable TBI outcomes.

## Figures and Tables

**Table 1:** Inclusion and exclusion criteria for CAPTAIN—II trial.

*Inclusion Criteria*	*Exclusion Criteria*
18 to 80 years	Presence of polytrauma (a score for the Abbreviated Injury Scale of >2 for other body regions)
Provided informed consent	Presence of spinal cord injury
Willingness to follow protocol requirements	Prior intracranial interventional/inter ischemic stroke and prior hemorrhagic stroke
The ability to communicate (verbally and in writing) and to read before TBI diagnosis	Psychiatric disorders
Glasgow Coma Scale (GCS) score between 7 and 12 for both hospital admission and pre-treatment	Neurodegenerative diseases
A score of ≤2 for other body regions	Prior epileptic seizure(s)
A score of 100 for the pre-trauma Karnofsky Index	Concomitantly medicated for previous cognitive impairments with cholinesterase inhibitors or neuroprotective agents
Marshal classification (CT) of one to six	Chronic diseases/disorders treated with cortisone, nootropic molecules or antipsychotic cortisone, Ca^+^ channel blockers, and antidepressants
Willingness to use birth control methods Negative result for pregnancy	Lack of informed consent and inability to comply with the study evaluations
Treatment initiation of up to four hours	Investigator assessment regarding study compliance

**Ca^+^** = calcium electrolytes, CT = computed tomography.

**Table 2:** Frequency of assessments within the three study visits.

Days—Neuropsychological Assessment	Neuropsychological Scales
10, 30, 90	PSI (Processing Speed Index, Wechsler adult intelligence scale—third edition), Stroop Color–Word Test—Victoria Version (VST), Mini-Mental State Examination (MMSE) [22,23,24]
30, 90	Color Trails Test, Digit Span (Wechsler adult intelligence scale), Hospital Anxiety and Depression Scale, Finger Tapping Test [25,26,27,28]

**PSI** = Processing Speed Index, **MMSE** = Mini-Mental State Examination.

**Table 3:** Study hypotheses for the Mann–Whitney U test.

Hypothesis—Type	Hypothesis—Statement
Null	The distributions of anxiety/depression subscales scores for Cerebrolysin (treatment) and saline solution (procedural placebo) are equal at the second/third study visit
Alternative	The distributions of anxiety/depression subscales scores for Cerebrolysin (treatment) and saline solution (procedural placebo) are not equal at the second/third study visit

**Table 4:** TBI patients’ age.

Age Group	*n* TBI Patients	% TBI Patients
18–20	4	3.2
21–30	24	19.2
31–40	33	26.4
41–50	14	11.2
51–60	23	18.4
61–70	15	12
71–79	12	9.6

***n***= crude number, **%** = percentage.

**Table 5:** Means and SDs of outcome scales across study visits and treatment groups.

	Cerebrolysin			Placebo (Saline Solution)		
Scale	Visit 1	Visit 2	Visit 3	Visit 1	Visit 2	Visit 3
**MMSE**	25.95 (SD: 3.3)	28.66 (SD:1.9)	29 (SD: 1.9)	25.93 (SD: 3.9)	28.19 (SD: 2.6)	29.02 (SD: 1.5)
**Stroop C-D**	25.41 (SD: 12.4)	24.72 (SD: 13)	23.3. (SD: 14.3)	25.6 (SD: 13.9)	24.72 (SD: 14.3)	24.28 (SD: 14.2)
**Stroop W-D**	8.45 (SD: 4.7)	7.51 (SD: 4.5)	6.03 (SD: 4.5)	10.6 (SD: 5.2)	9.76 (SD:4.9)	8.96 (SD: 4.6)
**PSI**	31.5 (SD: 10.3)	37.2 (SD:9.7)	41 (SD: 10.3)	30 (SD: 9.5)	35.77 (SD: 9.7)	37.63 (SD: 10.1)
**FFT Left**		41.11 (SD: 11.6)	43.4 (SD: 11.6)		42.6 (SD: 11.9)	49.64 (SD: 11.6)
**FFT Right**		45.34 (SD: 11.2)	48.28 (SD: 11.3)		46.8 (SD: 12.2)	44.09 (SD: 11.65)
**Digit Span Forward**		10.9 (SD: 2.7)	11.96 (SD: 2.5)		9.7 (SD: 3.1)	10.87 (SD: 2.9)
**Digit Span Backward**		8.27 (SD: 2.7)	9.34 (SD: 2.8)		6.94 (SD: 2.9)	7.63 (SD: 2.9)
**Color Trails 1**		76.3 (SD: 29.5)	68.9 (SD: 27.8)		84.4 (SD: 32.2)	79.72 (SD: 29)
**Color Trails 2**		124.63 (SD:50.9)	116.83 (SD: 49.9)		141.6 (SD: 63.4)	133.94 (SD: 62.7)
**HADS-Anxiety**		7.18 (SD: 3.7)	6.15 (SD: 3.7)		7.94 (SD: 4.9)	6.57 (SD: 4.8)
**HADS-Depression**		5.7 (SD: 3.5)	5.7 (SD: 3.5)		7.28 (SD: 4.5)	7.63 (SD: 4.8)

**SD** = Standard deviation, **MMSE** = Mini-Mental State Exam, **Stroop C** = naming time color words, **Stroop D** = naming time color dots control position (D), **Stroop W** = naming time common, **PSI** = Processing Speed Index, **FFT Left** = Finger Tapping Test, **FFT Right** = Finger Tapping Test Right, **HADS** = Hospital Anxiety and Depression Scale, **Color Trails 1** = Color Trails Test 1, **Color Trails 2** = Color Trails Test 2.

**Table 6:** Correlations with HADS-Anxiety.

Scales	Cerebrolysin Group				Placebo (Saline Solution)			
	*Visit 2*		*Visit 3*		*Visit 2*		*Visit 3*	
	*p-Value*	*r_s_*	*p-Value*	*r_s_*	*p-Value*	*r_s_*	*p-Value*	*r_s_*
**MMSE**	0.289	−0.177	0.710	0.077	*0.000*	−0.568	0.460	−0.162
**Stroop C-D**	*0.004*	0.341	0.091	0.202	*0.000*	0.507	*0.000*	0.516
**Stroop W-D**	*0.045*	0.239	*0.016*	0.286	0.052	0.265	0.281	0.149
**PSI**	*0.000*	−0.437	*0.018*	−0.279	*0.000*	−0.461	*0.002*	−0.415
**FFT Left**	*0.000*	−0.512	*0.000*	−0.410	*0.000*	−0.544	*0.000*	−0.501
**FFT Right**	*0.000*	−0.544	*0.005*	−0.330	*0.000*	−0.564	*0.000*	−0.507
**Digit Span Forward**	*0.008*	−0.313	0.194	−0.156	*0.000*	−0.620	*0.000*	−0.526
**Digit Span Backward**	*0.006*	−0.324	0.422	−0.097	*0.000*	−0.651	*0.000*	−0.565
**Color Trails 1**	*0.002*	0.360	0.209	0.151	*0.000*	−0.716	*0.000*	−0.593
**Color Trails 2**	*0.003*	0.351	*0.036*	0.250	*0.000*	0.629	*0.000*	0.494

***r_s_*** = rh**o**, **MMSE** = Mini-Mental State Exam, **Stroop C** = naming time color words, **Stroop D** = naming time color dots control position (D), **Stroop W** = naming time common, **PSI** = Processing Speed Index, **FFT Left** = Finger Tapping Test Left, **FFT Right** = Finger Tapping Test Right, **HADS** = Hospital Anxiety and Depression Scale, **Color Trails 1** = Color Trails Test 1, **Color Trails 2** = Color Trails Test 2.

**Table 7:** Correlations with HADS-Depression.

Scales	Cerebrolysin Group				Placebo (Saline Solution)			
	*Visit 2*		*Visit 3*		*Visit 2*		*Visit 3*	
	*p-Value*	*r_s_*	*p-Value*	*r_s_*	*p-Value*	*r_s_*	*p-Value*	*r_s_*
**MMSE**	0.055	−0.314	0.557	−0.121	*0* *.000*	−0.580	0.331	−0.212
**Stroop C-D**	*0* *.002*	0.367	*0* *.044*	0.240	*0* *.000*	0.499	0.089	0.234
**Stroop W-D**	0.231	0.144	*0* *.003*	0.352	0.047	0.272	0.088	0.234
**PSI**	*0* *.000*	−0.558	*0* *.000*	−0.575	*0* *.000*	−0.466	*0* *.000*	−0.549
**FFT Left**	*0* *.000*	−0.552	*0* *.000*	−0.549	*0* *.000*	−0.604	*0* *.001*	−0.455
**FFT Right**	*0* *.000*	−0.513	*0* *.000*	−0.500	*0* *.000*	−0.626	*0* *.000*	−0.526
**Digit Span Forward**	*0* *.000*	−0.510	*0* *.002*	−0.368	*0* *.000*	−0.646	*0* *.020*	−0.315
**Digit Span Backward**	*0* *.000*	−0.455	*0* *.002*	−0.363	*0* *.000*	−0.612	*0* *.045*	−0.273
**Color Trails 1**	*0* *.000*	0.522	*0* *.000*	0.485	*0* *.000*	−0.669	*0* *.012*	−0.339
**Color Trails 2**	0.000	0.532	0.000	0.542	0.000	0.534	0.049	0.270

***r_s_*** = rh**o, MMSE** = Mini-Mental State Exam, **Stroop C** = naming time color words, **Stroop D**—naming time color dots control position (D), **Stroop W** = naming time common, **PSI** = Processing Speed Index, **FFT Left** = Finger Tapping Test Left, **FFT Right** = Finger Tapping Test Right, **HADS** = Hospital Anxiety and Depression Scale, **Color Trails 1** = Color Trails Test 1, **Color Trails 2** = Color Trails Test 2.

## Data Availability

The data presented in this study were obtained from the CAPTAIN II study and are available upon reasonable request from the corresponding author.
